# High-throughput quantitation of amino acids and acylcarnitine in cerebrospinal fluid: identification of PCNSL biomarkers and potential metabolic messengers

**DOI:** 10.3389/fmolb.2023.1257079

**Published:** 2023-10-31

**Authors:** Jingjing Ma, Kun Chen, Yun Ding, Xiao Li, Qiming Tang, Bo Jin, Ruben Y. Luo, Sheeno Thyparambil, Zhi Han, C. James Chou, Ashlee Zhou, James Schilling, Zhiguang Lin, Yan Ma, Qing Li, Mengxue Zhang, Karl G. Sylvester, Seema Nagpal, Doff B. McElhinney, Xuefeng B. Ling, Bobin Chen

**Affiliations:** ^1^ Department of Hematology, Huashan Hospital, Fudan University, Shanghai, China; ^2^ Department of Laboratory Medicine, Huashan Hospital, Fudan University, Shanghai, China; ^3^ mProbe Inc., Palo Alto, CA, United States; ^4^ Department of Pathology, Stanford University School of Medicine, Stanford, CA, United States; ^5^ Department of Biomedical Data Science, Stanford University School of Medicine, Stanford, CA, United States; ^6^ Department of Surgery, Stanford University School of Medicine, Stanford, CA, United States; ^7^ Department of Neurology and Neurological Sciences, Stanford University School of Medicine, Stanford, CA, United States; ^8^ Departments of Cardiothoracic Surgery and Pediatrics (Cardiology), Stanford University School of Medicine, Stanford, CA, United States

**Keywords:** PCNSL, cerebrospinal fluid, amino acid, acylcarnitine, UHPLC-MS/MS

## Abstract

**Background:** Due to the poor prognosis and rising occurrence, there is a crucial need to improve the diagnosis of Primary Central Nervous System Lymphoma (PCNSL), which is a rare type of non-Hodgkin’s lymphoma. This study utilized targeted metabolomics of cerebrospinal fluid (CSF) to identify biomarker panels for the improved diagnosis or differential diagnosis of primary central nervous system lymphoma (PCNSL).

**Methods:** In this study, a cohort of 68 individuals, including patients with primary central nervous system lymphoma (PCNSL), non-malignant disease controls, and patients with other brain tumors, was recruited. Their cerebrospinal fluid samples were analyzed using the Ultra-high performance liquid chromatography - tandem mass spectrometer (UHPLC-MS/MS) technique for targeted metabolomics analysis. Multivariate statistical analysis and logistic regression modeling were employed to identify biomarkers for both diagnosis (Dx) and differential diagnosis (Diff) purposes. The Dx and Diff models were further validated using a separate cohort of 34 subjects through logistic regression modeling.

**Results:** A targeted analysis of 45 metabolites was conducted using UHPLC-MS/MS on cerebrospinal fluid (CSF) samples from a cohort of 68 individuals, including PCNSL patients, non-malignant disease controls, and patients with other brain tumors. Five metabolic features were identified as biomarkers for PCNSL diagnosis, while nine metabolic features were found to be biomarkers for differential diagnosis. Logistic regression modeling was employed to validate the Dx and Diff models using an independent cohort of 34 subjects. The logistic model demonstrated excellent performance, with an AUC of 0.83 for PCNSL vs. non-malignant disease controls and 0.86 for PCNSL vs. other brain tumor patients.

**Conclusion:** Our study has successfully developed two logistic regression models utilizing metabolic markers in cerebrospinal fluid (CSF) for the diagnosis and differential diagnosis of PCNSL. These models provide valuable insights and hold promise for the future development of a non-invasive and reliable diagnostic tool for PCNSL.

## 1 Introduction

Primary central nervous system lymphoma (PCNSL) is a rare and highly malignant type of non-Hodgkin’s lymphoma that predominantly affects the brain, cerebrospinal fluid (CSF), intraocular structures, and spinal cord (Wang et al., 2014). It accounts for approximately 2%–3% of all brain tumors (DeWitt et al., 2017), with an incidence of 0.47/100,000, and has shown an increasing incidence in recent years, primarily among the elderly and immunocompromised populations (Villano et al., 2011). The diagnosis of PCNSL poses challenges due to its varied clinical manifestations, which depend on the specific region of the central nervous system involved (Baraniskin and Schroers, 2021). Currently, the diagnostic process for PCNSL typically involves a combination of imaging techniques such as magnetic resonance imaging (MRI) and computed tomography (CT) scan (Ota, 2021), along with biopsy or other tissue sample analysis. However, MRI findings may not be specific enough to distinguish PCNSL from other conditions such as glioma, intramural infection, or non-infectious inflammation (Bataille et al., 2000). Pathological examination remains the gold standard for diagnosing PCNSL (Eichler and Batchelor, 2006; Matsuzono et al., 2021). Biopsy, although considered the gold standard for diagnosis, carries potential risks of complications such as bleeding, infection, or neurological damage, especially when the tumor is located in a sensitive area of the brain. Additionally, biopsies can yield false negative results due to inadequate tissue sampling or prior use of hormones (van Westrhenen et al., 2018). In contrast, obtaining CSF samples is a less traumatic procedure that can provide valuable insights into the composition of the extracellular fluid within the central nervous system. Therefore, there is a critical need to develop an accurate and minimally invasive diagnostic method based on CSF analysis to improve the detection of PCNSL.

In recent years, metabolomics has gained popularity as a powerful tool in medicine and life sciences. It can identify and measure small molecule metabolites in biological samples, helping to elucidate the underlying pathological mechanisms of diseases by exploring the relationships between metabolites (Beger, 2013; Deja et al., 2014; Qi et al., 2021). Amino acids and acylcarnitines are essential metabolites that provide energy for the body and brain and their circulating levels serve as indicators of metabolic disorders. In recent years, metabolomic analysis of amino acids and acylcarnitines has shown effectiveness in diagnosing cancer. Studies have demonstrated that specific amino acids and acylcarnitines, such as valine and leucine, play a role in multiple cancer phenotypes and act as markers of disease pathology (S et al., 2020). For example, Yao et al. reported significant differences in levels of several amino acids and acylcarnitines between individuals with papillary thyroid carcinoma and healthy controls (Yao et al., 2011). Ni et al. demonstrated that a PLS-DA model based on glycine, valine, methionine, citrulline, arginine, and C16-carnitine exhibited a strong ability to distinguish patients with lung cancer from healthy controls (Ni et al., 2019). Zhang et al. reported that leucine, isoleucine, and valine levels were significantly upregulated in the serum of breast cancer patients compared to healthy donors (Zhang and Han, 2017). Zhou et al. found that two long-chain acylcarnitines were significantly higher in cirrhosis and hepatocellular carcinoma patients compared to healthy controls (Zhou et al., 2012).

As for the brain tumor, metabolomic profiling of CSF also reveals a great potential for discoveries of novel biomarkers for disease screening and diagnosis. Because CSF directly interacts with the tissue of the central nervous system (CNS) and is readily accessible with less-invasive procedures, it provides an attractive source of useful markers for clinical diagnostics. This was supported by findings from our lab and other groups. Thirty-nine metabolites have been identified with significant changes in the CSF of the malignant gliomas relative to the control samples (Locasale et al., 2012). Wang et al. identified significant changes in glutamine and butyryl carnitine levels between PCNSL and healthy control samples (Wang et al., 2020). Using targeted metabolomics, we developed a logical regression model based on six metabolic markers in CSF that can effectively predict PCNSL patient prognosis before the HD-MTX-based chemotherapy treatments (Zhou et al., 2023).

However, the investigation of amino acid and acylcarnitine levels in the cerebrospinal fluid (CSF) of PCNSL patients using targeted metabolomics techniques has been limited in existing studies. To explore the potential association between amino acid and acylcarnitine levels in CSF and PCNSL, we employed a comprehensive targeted metabolomic approach. This method allowed us to quantify 20 amino acids and 25 acylcarnitines, aiming to identify distinctive metabolic patterns specific to primary central nervous system lymphoma (PCNSL). Through the application of this advanced assay, we successfully developed and validated biomarker panels capable of accurately discerning PCNSL patients from individuals with non-malignant diseases and other brain tumor patients.

## 2 Materials and methods

### 2.1 Human CSF sample collection and ethics

CSF samples from both patients and healthy individuals were obtained from the departments of neurosurgery, neurology, infection, and hematology at Huashan Hospital of Fudan University in Shanghai, China. The collection of CSF followed established protocols, with samples being collected before any treatment was administered. The procedure involved lumbar puncture at room temperature, with the initial 10 drops of CSF being discarded to prevent blood contamination. After collection, the CSF samples were centrifuged at 3,000 rpm for 10 min to remove cellular debris. The resulting supernatant was divided into 1 mL aliquots and stored at −80°C until analysis. The study received approval from the Ethics Committee of Huashan Hospital of Fudan University, and all procedures adhered to the principles outlined in the Declaration of Helsinki and Good Clinical Practice guidelines. Informed consent was obtained from all participants prior to their involvement in the study.

### 2.2 Chemicals and reagents

The Labeled amino acid mix, labeled acylcarnitine mix, and labeled acylcarnitine mix supplement used in the study were obtained from Cambridge Isotope Laboratories Inc. (Andover, MA). LC-MS grade methanol, ammonium formate, water and formic acid were obtained from Thermo-Fisher Scientific (FairLawn, NJ). HPLC grade Hexane was obtained from Fisher Scientific (FairLawn, NJ). The GC grade beta-mercaptoethanol was purchased from Sigma (Buchs, Sweitzerland).

### 2.3 CSF sample preparation

To prepare the CSF sample for analysis, a 10 μL aliquot of the CSF sample was spiked with 10 μL of an internal standard working solution. The internal standard working solution contained 25 μM of ^13^C_5_, ^15^N-Proline (CNLM-436), isotope labeled Amino Acid Mix (NSK-A), Acylcarnitine Mix (NSK-B), and Acylcarnitine Mix Supplement (NSK-B-G).

The mixture was then subjected to extraction using 90 μL of extraction buffer (1‰ HCL and 1% beta-mercaptoethanol in methanol) and 200 μL of hexane. After vigorous vortexing for 60 s, the mixture was centrifuged at 12,000 g for 5 min. The hydrophilic amino acids and acylcarnitines were extracted into bottom methanol layer and the lipophilic molecules were extracted into up hexane layer and discarded. Subsequently, 80.00 μL of the lower layer was transferred into an auto-sampler vial for further analysis using UHPLC-MS/MS.

### 2.4 LC−MS analysis

Following the CSF sample preparation, a 2 µL extract was injected into the Vanquish UHPLC system (Thermo Fisher, Germering, Germany). Isocratic elution was performed using a mobile phase A composed of water with 0.5% formic acid and 10 mM ammonium formate, and a mobile phase B consisting of methanol with 0.1% formic acid and 10 mM ammonium formate. The elution was carried out with a constant flow rate of 0.1 mL/min, with 50% mobile phase B. The metabolites were detected using an Altis mass spectrometer (Thermo Fisher, San Jose, CA, United States) operating in MRM (Multiple Reaction Monitoring) mode. The specific MRM transitions for the targeted amino acids and acylcarnitines can be found in Supplementary Tables S1, S2. The Q1 and Q3 resolutions were both set at 0.7 Da, and the cycle time was set to 0.8 s. The spray voltage was optimized at 3500 V, and the gas flows were adjusted to 20, 5, and 0 Arb for Sheath Gas, Aux Gas, and Sweep Gas, respectively. The ion transfer tube was maintained at a temperature of 300°C, and the vaporizer temperature was set at 200°C.

### 2.5 Quantitation

The concentration quantitation of individual targeted amino acdis and acylcarnitines was implemented by multiplying the internal standard concentration with the peak area ratio between the analytes and the corresponding internal standard. The relationship for quantitation is detailed in Supplementary Tables S3, S4.

### 2.6 Statistical analysis

Five steps of statistical analysis were followed to determine the final panels for diagnosis and differentiation of PCNSL from non-malignant and other brain tumor patients.

Feature generation-Features used for univariant analysis included two sets, 45 individual amino acids and acylcarnitine concentrations and 1,642 ratios. Individual concentrations were quantified based on the above quantification method and ratios were calculated using all different combinations with either 2 individual analyte’s concentrations. Complete features were listed in Supplementary Tables S5.

Univariant analysis-Univariate analysis was performed between PCNSL and non-malignant controls and between PCNSL and other brain tumor patients. Fold change (medium value) and an AUC ROC (Area Under the Receiver Operating Characteristic Curve) of each feature were calculated for following feature selection.

Feature selection-Significant differential features between groups were selected based on an AUC ROC greater than 0.8 and a |log2 fold change| greater than 0.26. A reverse ablation analysis using logistic coefficients was performed for significant differential features to select a minimum feature panel with highest classification performance (highest AUCROC).

Multivariate modeling-Four multivariate modeling algorithms, Logistic Regression (LR), Lasso, Elastic Net (EN) and K-Nearest Neighbors (KNN), were compared to evaluate the performance of classifying PCNSL patients from other groups.

Validation- In this study, a cohort of 33 PCNSL patients, 33 other brain tumor patients, and 36 non-malignant disease control participants were divided into training (n = 68) and validation (n = 34) sets. Model developed from training sets was tested in the validation set and classification model AUCROC was evaluated. Homemade R script was utilized for feature selection and model fitting processes in this study. We utilized the logistic regression implementation available in the ‘glm’ package, Elastic Net (EN) model was implemented using the ‘glmnet’ package and the KNN model was implemented using the ‘caret’ package. Specifically, hyperparameter tuning for EN and KNN model was conducted using the entire dataset. The performance of model (AUCROC) was calculated using ‘pROC’ package.

## 3 Results

### 3.1 Study design

**FIGURE 1:**
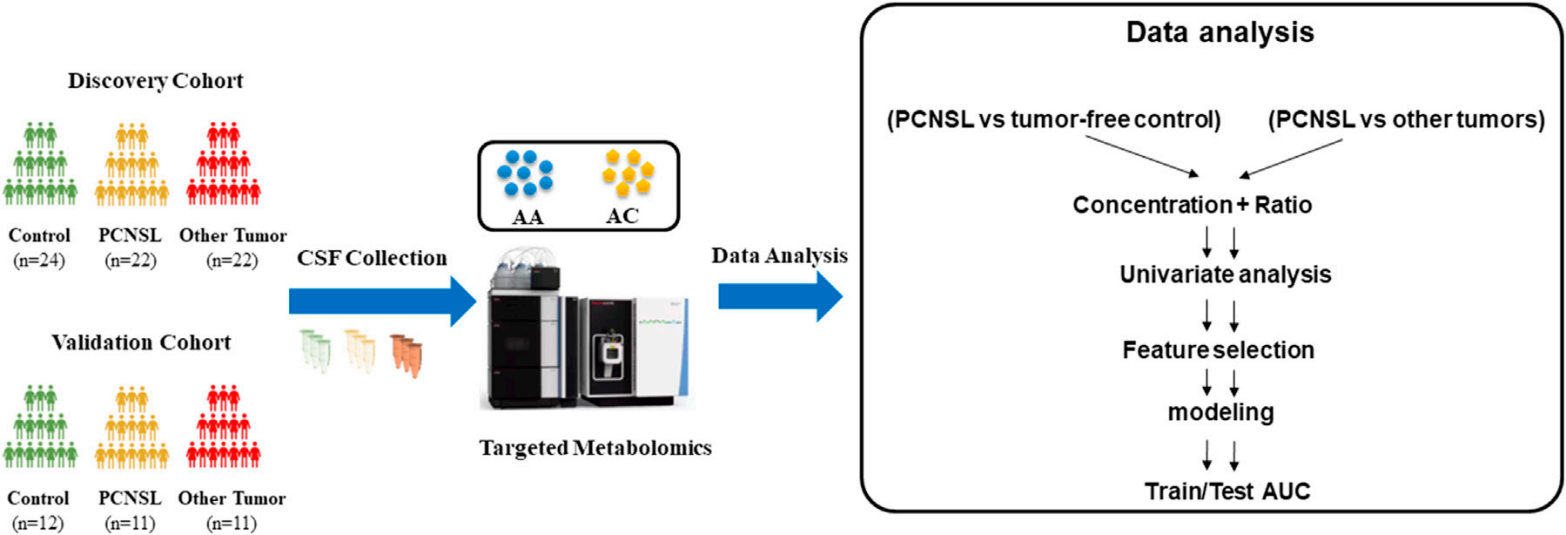
Study workflow diagram.

The overall study workflow was depicted in Figure 1. First, CSF samples were collected and categorized into three groups: PCNSL patients (PCNSL Group), patients with other brain tumor cancers (Other Brain Tumor Group), and control patients with non-malignant diseases (Control Group). Each group’s samples were randomly divided into discovery and validation sets at a ratio of 2:1. Second, quantification of 45 amino acids and acylcarnitines was performed for all CSF samples. To expand the feature space, concentration ratios of two metabolites were calculated in addition to using the concentration of a single metabolite as a feature. As the third step, the metabolic changes between the PCNSL Group and the Control Group, as well as the Other Brain Tumor Group, were compared and analyzed in the discovery sets. Finally, the discovered metabolite biomarkers were independently tested, and models were constructed using the validation sets.

### 3.2 Cohort collection and targeted metabolism in CSF

In this study, a total of 102 individuals were collected, consisting of 33 PCNSL patients, 36 non-malignant disease controls, and 33 other brain tumor patients. Baseline demographic and clinical data, including sex, age, CSF nucleated cells, CSF protein, CSF tumor cells, CSF IL-10, CSF IL-6, and brain parenchyma lesion, were recorded and summarized in Table 1 and Table 2. The samples were randomly divided into a discovery group, comprising two-thirds of the samples, and a validation group, comprising the remaining one-third. In the diagnosis study (Table 1), the mean age of PCNSL patients in the discovery group was 59 years ears, with 14 males and 8 females, while the non-malignant disease control group had a mean age of 47.5 years, with 11 males and 13 females. In the validation group, the mean age of PCNSL patients was 52 years, with 7 males and 4 females, while the non-malignant disease control group had a mean age of 53.5 years, with 4 males and 8 females. In the differential diagnosis study (Table 2), the mean age of PCNSL patients in the discovery group was 54.5 years, with 15 males and 7 females, while the other brain tumor patients had a mean age of 57.5 years, with 6 males and 16 females. In the validation group, the mean age of PCNSL patients was 57 years, with 6 males and 5 females, while the other brain tumor patients had a mean age of 63 years, with 6 males and 5 females. No significant differences in age and gender were found between the PCNSL and other brain tumor groups or between the PCNSL and tumor-free control groups.

**TABLE 1:** Demographics table of PCNSL Dx.

Characteristic	Train		Test	
	PCNSL (n = 22)	Control (n = 24)	PCNSL (n = 11)	Control (n = 12)
Age				
Median (range)	59 (26–80)	47.5 (19–70)	52 (33–78)	53.5 (19–72)
Sex				
Male	14	11	7	4
Female	8	13	4	8
CSF nucleated cells				
(0–8) × 10^9^/L	15	18	8	12
>8 × 10^9^/L	7	6	3	0
CSF protein				
>0.45 g/L	12	11	9	4
≤0.45 g/L	10	13	2	8
CSF tumor cells				
Positive	13	1	8	0
Negative	9	23	3	12
CSF IL-10				
Evaluated	15	ND	8	ND
Normal	7	ND	3	ND
CSF IL-6				
Evaluated	15	ND	8	ND
Normal	7	ND	3	ND
Brain parenchyma lesion				
Yes	21	11	11	5
No	1	13	0	7

*ND: Not Determined (Not Tested).

**TABLE 2:** Demographics table of PCNSL Diff.

Characteristic	Train		Test	
	PCNSL (n = 22)	Control (n = 22)	PCNSL (n = 11)	Control (n = 11)
Age				
Median (range)	54.5 (26–80)	57.5 (34–69)	57 (33–78)	63 (39–78)
Sex				
Male	15	13	6	6
Female	7	9	5	5
CSF nucleated cells				
(0–8) × 10^9^/L	17	13	6	10
>8 × 10^9^/L	5	9	5	1
CSF protein				
>0.45 g/L	13	15	8	7
≤0.45 g/L	9	7	3	4
CSF tumor cells				
Positive	14	14	7	6
Negative	8	8	4	5
CSF IL-10				
Evaluated	16	0	7	0
Normal	6	22	4	11
CSF IL-6				
Evaluated	16	8	7	5
Normal	6	14	4	6
Brain parenchyma lesion				
Yes	21	21	11	9
No	1	1	0	2

### 3.3 CSF metabolite biomarker distinguishing PCNSL from non-malignant cancers

**FIGURE 2:**
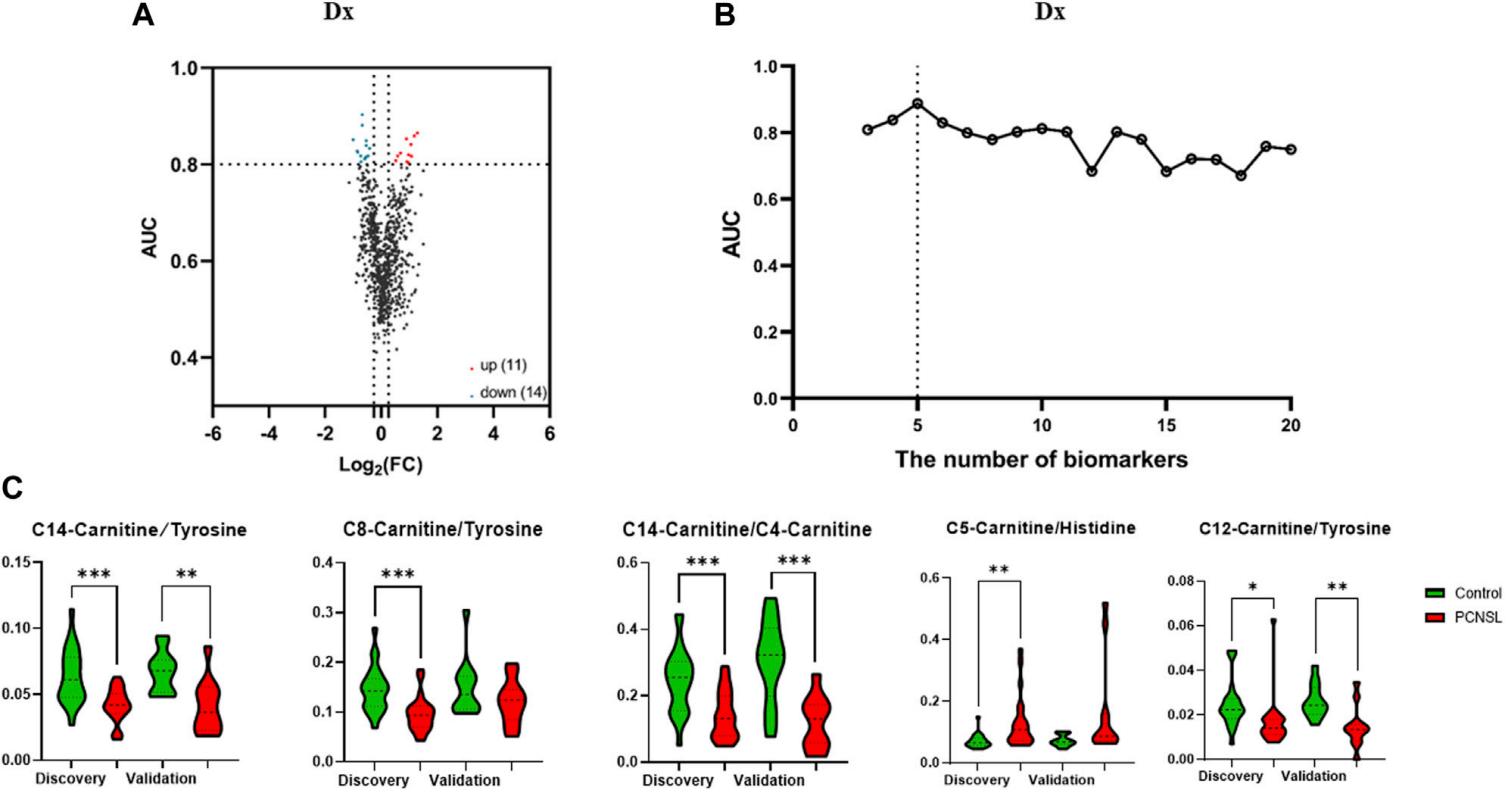
The selection of 5 specific biomarkers for PCNSL diagnosis. **(A)** The volcano plot of 45 metabolite and their ratios, and 25 significant changed components (|log2 fold change| > 0.26, AUC ROC >0.8) for PCNSL diagnosis and **(B)** Logistic model performance using the top 20 high coefficient features. **(C)** The violin plot of 5 validated metabolic biomarkers for PCNSL diagnosis.

In order to investigate the potential link between CSF levels of amino acids and acylcarnitines and PCNSL, we conducted a comprehensive targeted metabolomic analysis using LC-MS/MS. We quantified a total of 18 amino acids and 26 acylcarnitines in the CSF samples obtained from PCNSL patients, controls with non-malignant diseases, and other brain tumor patients. To enhance the scope of our analysis, we expanded the number of features of interest by not only considering the direct concentration measurements of the 45 metabolites, but also incorporating the ratios of any two metabolite combinations as new features. This resulted in a total of 1,682 features. As depicted in Figure 2A, we identified 25 features that exhibited an AUC ROC (Area Under the Receiver Operating Characteristic Curve) greater than 0.8 and a |log2 fold change| greater than 0.26, indicating significant alterations in metabolite levels.

To determine the most effective combination of features for classification, we conducted a reverse ablation analysis using the logistic coefficient of each feature. Based on this analysis, we selected the top 20 features with the highest coefficients and evaluated the logistic model performance. We found that the minimal feature panel consisting of the top 5 important features yielded the highest classification performance in the discovery set. These top 5 features were C14-Carnitine/Tyrosine, C8-Carnitine/Tyrosine, C14-Carnitine/C4-Carnitine, C5-Carnitine/Histidine, and C12-Carnitine/Tyrosine, as illustrated in Figure 2B. To validate the robustness of these features, we further examined their performance in the validation set. Remarkably, all 5 features exhibited significant differences in at least one dataset, as demonstrated in Figure 2C. These findings highlight the potential of these features as reliable biomarkers for classification purposes.

**FIGURE 3:**
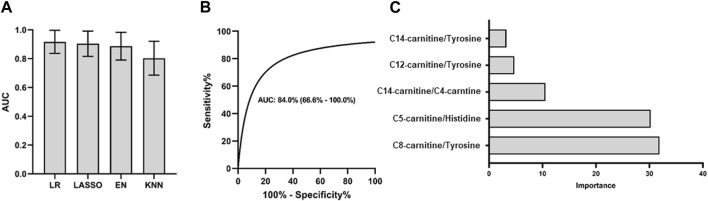
PCNSL diagnosis panel with 5 validated metabolic biomarkers. Different models are used for **(A)** PCNSL diagnosis with discovery cohort. ROC curve for the validation model with the best AUC performance in **(B)** and the coefficient importance of 5 validated metabolic biomarkers for PCNSL diagnosis in **(C)**. LR: Logistic regression; Lasso: Least Absolute Shrinkage and Selection Operator; EN: Elastic Net linear regression; KNN: K-Nearest Neighbors.

We conducted diagnostic model testing using 5 ratios in 4 different models, and the logistic model demonstrated the best performance for PCNSL diagnosis in the discovery set, as depicted in Figure 3A. To validate the effectiveness of this model, we further evaluated its performance on the validation cohort. The model exhibited favorable results with an AUC ROC of 0.84 (95%CI: 0.67–1.00), high specificity (0.82), and sensitivity (0.83), as shown in Figure 3B. Among the metabolite ratios included in the model, C8-Carnitine/Tyrosine and C5-Carnitine/Histidine made the most significant contributions, collectively accounting for over 60% of the combined coefficient, as illustrated in Figure 3C. These findings suggest that the top 5 ratios, particularly C8-Carnitine/Tyrosine and C5-Carnitine/Histidine, hold promise as potential CSF biomarkers for the classification and diagnosis of PCNSL.

### 3.4 CSF metabolite biomarker distinguishing PCNSL from other brain tumor cancers

**FIGURE 4:**
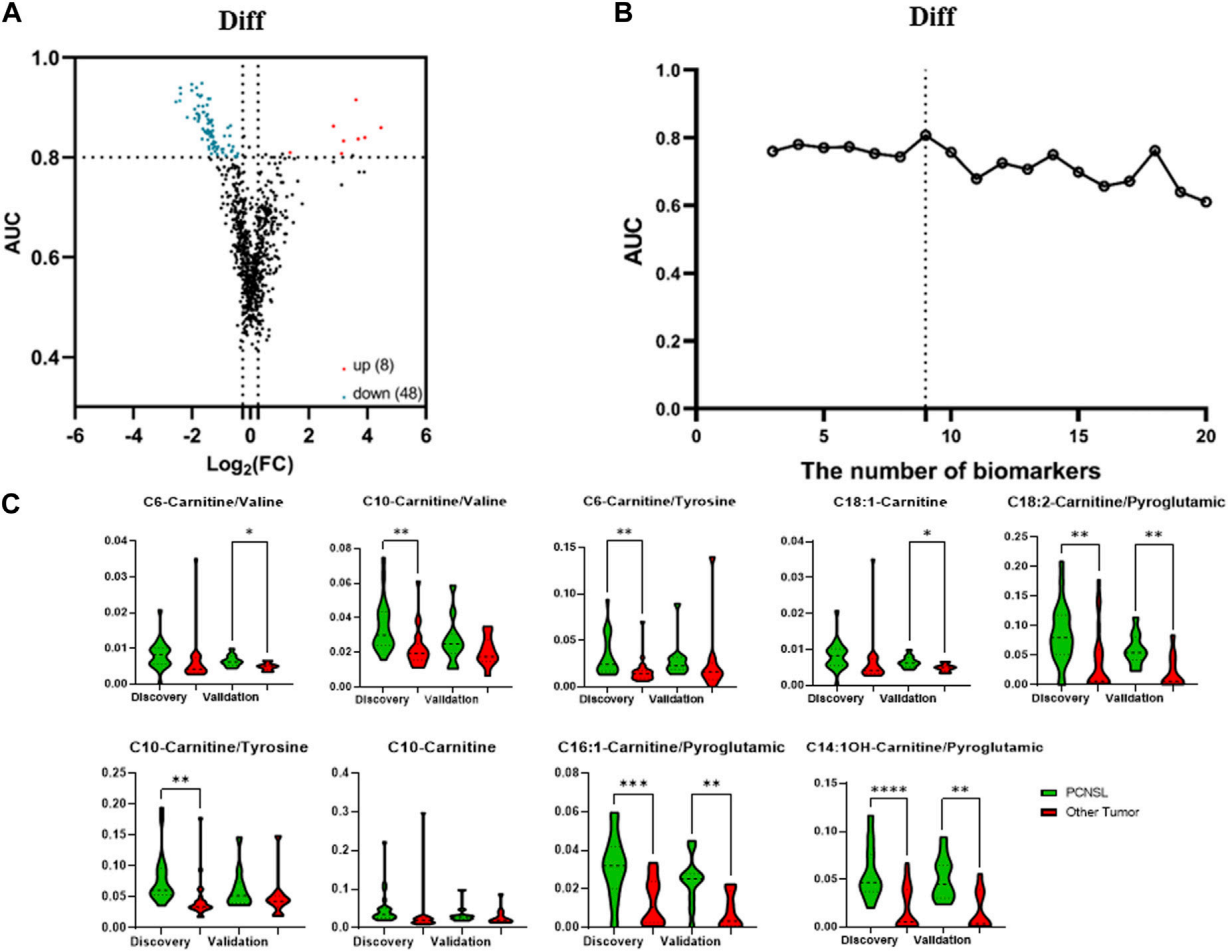
The selection of 9 specific biomarkers for PCNSL differential diagnosis. **(A)** The volcano plot of 45 metabolite and their ratios, and **(B)** Logistic model performance using the top 20 high coefficient features. **(C)** The violin plot of 9 validated metabolic biomarkers for PCNSL differential diagnosis.

The analysis pipeline and criteria for differentially diagnosing PCNSL from other brain tumor cancers were similar to those used for diagnosing PCNSL from non-malignant diseases. In our univariate analysis, as illustrated in Figure 4A, a total of 56 features were identified that met the criteria of having a |log2 fold change| > 0.26 and an AUC ROC greater than 0.8 for the differential diagnosis of PCNSL from other brain tumor cancers. We then evaluated the performance of different feature panels, containing a maximum of the top 20 coefficients in the logistic model, and observed that the panel comprising the top 9 features exhibited the best performance on the discovery set, as shown in Figure 4B. These 9 features consisted of 2 individual metabolites (C18:1-Carnitine and C10-Carnitine) and 7 ratios (C6-Carnitine/Valine, C10-Carnitine/Valine, C6-Carnitine/Tyrosine, C18:2-Carnitine/Pyroglutamic, C10 Carnitine/Tyrosine, C16:1-Carnitine/Pyroglutamic, and C14:1OH-Carnitine/Pyroglutamic, as depicted in Figure 4C.

**FIGURE 5:**
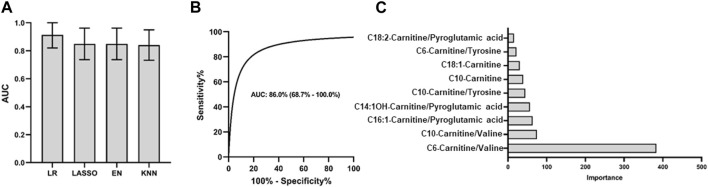
PCNSL differential diagnosis panel with 9 validated metabolic biomarkers. Different models are used for **(A)** PCNSL differential diagnosis with discovery cohort. ROC curve for the validation model with the best AUC performance in **(B)** and the coefficient importance of 9 validated metabolic biomarkers for PCNSL differential diagnosis in **(C)**. LR: Logistic regression; Lasso: Least Absolute Shrinkage and Selection Operator; EN: Elastic Net linear regression; KNN: K-Nearest Neighbors.

The four models used in the PCNSL diagnostic analysis were also applied to evaluate the performance of the nine metabolite features in the differential diagnosis of PCNSL from other brain tumor cancers. Consistent with the PCNSL diagnostic analysis, the logistic model exhibited the best performance on the discovery set, as shown in Figure 5A. The model’s performance was further validated on an independent cohort, where it demonstrated excellent performance with an AUC ROC of 0.86 (95%CI: 0.69–1.00), high specificity (0.82), and sensitivity (0.91), as depicted in Figure 5B. The logistic coefficient analysis indicated that C6-Carnitine/Valine was the most important feature in the model, as illustrated in Figure 5C. These findings suggest that the nine metabolite features identified in this study have the potential to serve as CSF biomarkers for distinguishing PCNSL from other brain tumor cancers.

## 4 Discussion

Over the past decade, there has been a significant amount of metabolomics research aimed at identifying reliable biomarkers for screening and diagnosing PCNSL using nuclear magnetic resonance and liquid chromatography-mass spectrometry techniques, resulting in extensive findings. Several types of human cancers, such as thyroid cancer (Yao et al., 2011), lung cancer (Ni et al., 2019), breast cancer (Zhang and Han, 2017), colorectal cancer (Farshidfar et al., 2018), endometrial cancer (Wang et al., 2018), bone sarcomas (Martin et al., 2020), and glioblastoma (Zhang et al., 2021), are shown to be accompanied by significant changes in amino acid catabolism, leading to detectable alterations in the amino acid levels found in body fluids. Similarly, several studies have reported changes in the metabolic levels of acylcarnitines in certain types of cancer, including thyroid cancer (Yao et al., 2011), lung cancer (Ni et al., 2019), colorectal cancer (Farshidfar et al., 2018), hepatocellular carcinoma (Zhou et al., 2012) and breast cancer (His et al., 2019) As for the tumor located in the central nervous system, Wang et al. utilized untargeted metabolomics to profile the metabolic signatures of different brain tumors with CSF samples, identifying metabolites mainly related to amino acid metabolism (Wang et al., 2020). Kim et al. revealed significant differences in NMR spectra of CSF between PCNSL and normal groups and developed a prediction model with 6 metabolites to diagnose PCNSL (Kim et al., 2023). In this study, we employed targeted metabolomic analysis with UHPLC-MS technique to determine the amino acid and acylcarnitine levels in patients with PCNSL. Additionally, we utilized logistic algorithm to select the most important markers, resulting in a DX model with sensitivity of 83%, specificity of 82%, and AUC of 0.84 and a Diff model with sensitivity of 91%, specificity of 82%, and AUC of 0.86, which were established for detecting PCNSL.

In our study, we identified specific metabolites and metabolite ratios that have the potential to serve as biomarkers for distinguishing primary central nervous system lymphoma (PCNSL) patients from non-malignant disease controls, as well as for the differential diagnosis of PCNSL from other brain tumor cancers. For the PCNSL *versus* non-malignant disease control comparison, the potential biomarkers included C14 Carnitine/Tyrosine, C8-Carnitine/Tyrosine, C14-Carnitine/C4-Carnitine, C5-Carnitine/Histidine, and C12-Carnitine/Tyrosine. For the differential diagnosis of PCNSL, the potential biomarkers comprised C10-Carnitine, C18:1-Carnitine, C6-Carnitine/Valine, C6-Carnitine/Tyrosine, C10 Carnitine/Valine, C10-Carnitine/Tyrosine, C14:1OH Carnitine/Pyroglutamic acid, C16:1-Carnitine/Pyroglutamic acid, and C18:2-Carnitine/Pyroglutamic acid. Detailed information and violin plots of these selected biomarkers are shown in Figure 2 and Figure 4 of our study. These findings contribute to the potential development of metabolite-based diagnostic approaches for PCNSL.

Amino acids, being the essential constituents of proteins, play vital roles in human metabolism, cell proliferation, gene expression, and inflammatory response. The proliferation of tumor cells relies heavily on the availability of amino acids for nitrogen supply, supporting protein and nucleotide biosynthesis (Ward and Thompson, 2012). This process can lead to the accumulation of reactive oxygen species (ROS) within cancer cells, causing damage to macromolecules and eventual cell death (Lieu et al., 2020) Changes in amino acid levels can significantly impact tumor cells and the immune microenvironment, resulting in altered amino acid profiles in cancer patients. Notably, glutamine, valine, leucine, isoleucine, and glycine have demonstrated their significance in cancer growth and metastasis (Hensley et al., 2013; Green et al., 2016). Valine, a branched-chain amino acid, participates in metabolism by generating succinyl-CoA and acetyl-CoA, which are then oxidized for energy production and fatty acid synthesis (Stine et al., 2022). Valine intake has been associated with tumor growth, as demonstrated in a mouse model with transplanted lymphocytic leukemia (Thandapani et al., 2022). Histidine, an essential acid that plays a vital role in human, especially in the myelin sheath and information transmission from the brain, influences tumor proliferation, invasion, metastasis, and overall prognosis, with its phosphorylation level playing a significant role (Dong et al., 2021). Tyrosine, an aromatic amino acid, has been identified as a potential biomarker for gastroesophageal cancer, with significantly lower concentrations observed in gastric cancer cases in both early and late disease stages (Wiggins et al., 2015). Pyroglutamic acid, an intermediate in glutathione metabolism, affects glutathione levels and oxidative stress (Gamarra et al., 2019). Glutathione homeostasis disruption is associated with tumor initiation and progression, as it plays a crucial role in cell differentiation, proliferation, apoptosis, ferroptosis, and immune function. Thus, pyroglutamic acid may have indirect effects on cancer. For instance, He et al. reported that serum pyroglutamic acid concentrations decreased from prostatic hyperplasia to prostate cancer (He et al., 2022). In addition to the individual amino acid, ratios of some amino acids also shows differential profiles in cancers. For instance, increased ratio of serum kynurenine/tryptophan was found to be correlated with lung cancer progression (Suzuki et al., 2010)and hepatitis B virus (HBV)-related hepatocellular carcinoma (Wu et al., 2022). In the same study of HBV-related hepatocellular carcinoma, branched-chain amino acids (BCAA)/tyrosine ratio was also shown to be significantly decreased. These findings suggest a strong correlation between cancer progression and amino acid profile (Fan et al., 2012) and are in line with our own research results.

Acylcarnitines, which are acylated derivatives of carnitine, play a crucial role in mitochondrial and peroxisomal transport for *β*-oxidation of fatty acids. Alterations in their levels can indicate disruptions in fatty acid oxidation, glycolysis, and branched-chain amino acid metabolism, providing valuable insights into cancer development and progression (Houten et al., 2020; McCann et al., 2021). The targeted measurement of acylcarnitines presents novel opportunities for cancer diagnosis and prognosis. For instance, Lu et al. employed LC-MS technology to analyze the acylcarnitine metabolic profile of hepatocellular carcinoma, observing significant changes in short-chain, medium-chain, and long-chain acylcarnitine levels within the cancer samples (Lu et al., 2019). Similar outcomes were reported by Bogusiewicz et al. in their study profiling acylcarnitines in gliomas (Bogusiewicz et al., 2021). In the context of breast cancer diagnosis, C12-Carnitine, C14-Carnitine, and C14:2-Carnitine have been identified as potential markers (Kozar et al., 2021), while His et al. found a positive association between plasma concentration of acylcarnitine (C2-Carnitine) and breast cancer risk (His et al., 2019). Additionally, Li et al. highlighted the diagnostic potential of elevated acylcarnitines, specifically C6-Carnitine, in distinguishing gastric cancer from control samples (Li et al., 2022). Ni et al. developed an accessible metabolomics-based early cancer detection method using LC-MS technology and discovered significantly increased levels of acylcarnitines, including C3-Carnitine, C4-Carnitine, C5-Carnitine, C14-Carnitine, and C16-Carnitine, in lung cancer patients compared to healthy controls. (Ni et al., 2016). Moreover, the ratios between different acylcarnitines can provide valuable metabolic information. Yao et al. reported a decreased ratio of palmitoyl-carnitine to carnitine (C16-Carnitine/C0-Carnitine) in the papillary thyroid carcinoma group, indicating carnitine palmitoyl transferase I activity (Yao et al., 2011). In another study, C5OH-Carnitine/C0-Carnitine, C3-Carnitine/Methionine, and Valine/Phenylalanine ratios were shown to be significant risk factors for hepatocellular carcinoma (Zhang et al., 2018). These findings suggest that the absolute amount or proportion of acylcarnitines may reflect the metabolic status and progression of cancer. In our study, we observed decreased ratios of C14-Carnitine/Tyrosine, C8-Carnitine/Tyrosine, C14-Carnitine/C4-Carnitine, and C12-Carnitine/Tyrosine, as well as an increased ratio of C5-Carnitine/Histidine in PCNSL patients compared to controls. In our differential diagnosis model, we identified nine features, including two individual metabolites (C18:1-Carnitine and C10-Carnitine) and seven ratios (C6-Carnitine/Valine, C10-Carnitine/Valine, C6-Carnitine/Tyrosine, C18:2-Carnitine/Pyroglutamic, C10-Carnitine/Tyrosine, C16:1-Carnitine/Pyroglutamic, and C14:1OH-Carnitine/Pyroglutamic), all of which were elevated in PCNSL compared to other brain tumor groups. These experimental findings underscore the significance of these acylcarnitines, amino acids, and their ratios as significant biomarkers for the diagnosis or differential diagnosis of PCNSL.

There are several limitations to our study that should be considered. Firstly, the sample size was relatively small, and larger studies are needed to confirm and generalize our findings. Additionally, we did not take into account other potential factors that could influence amino acid and acylcarnitine metabolism, such as the stage of cancer progression. Further research is necessary to explore these factors and their impact on the observed metabolic changes.

## Data Availability

The raw data supporting the conclusion of this article will be made available by the authors, without undue reservation.

## Abbreviations

PCNSL, Primary Central Nervous System Lymphoma; CSF, Cerebrospinal fluid; UHPLC-MS/MS, Ultra-high performance liquid chromatography - tandem mass spectrometer; MRI, magnetic resonance imaging; CT, computed tomography; AUC ROC, Area Under the Receiver Operating Characteristic Curve; LR, Logistic regression; Lasso, Least Absolute Shrinkage and Selection Operator; EN, Elastic Net linear regression; KNN, K-Nearest Neighbors.
