# Supplementary material for: High-throughput quantitation of amino acids and acylcarnitine in cerebrospinal fluid: identification of PCNSL biomarkers and potential metabolic messengers
Source: Front Mol Biosci. 2023 Oct 31;10:1257079. doi: 10.3389/fmolb.2023.1257079 (PMC10644155; doi:10.3389/fmolb.2023.1257079)
Supplement: Supplementary file 2 [file Table1.DOCX]

**Supplementary Table 1 The optimized transitions for amino acids.**

| **Compound** | **RT (min)** | **RT Window (min)** | **Polarity** | **Precursor (m/z)** | **Product (m/z)** | **Collision Energy (V)** | | **RF Lens (V)** |
| --- | --- | --- | --- | --- | --- | --- | --- | --- |
| Glycine | 0.45 | 0.9 | Positive | 76 | 30 | 10 | | 30 |
| Alanine | 0.45 | 0.9 | Positive | 90 | 44 | 10 | 30 | |
| Serine | 0.45 | 0.9 | Positive | 106 | 60 | 18 | 30 | |
| Proline | 0.45 | 0.9 | Positive | 116.1 | 70 | 17 | 32 | |
| Valine | 0.45 | 0.9 | Positive | 118.1 | 72 | 10 | 66 | |
| Threonine | 0.45 | 0.9 | Positive | 120.1 | 74 | 15 | 38 | |
| Pyroglutamic acid | 0.45 | 0.9 | Positive | 130.1 | 84 | 17 | 30 | |
| Leucine/Isoleucine | 0.45 | 0.9 | Positive | 132.1 | 86 | 10 | 30 | |
| Ornithine | 0.45 | 0.9 | Positive | 133.1 | 70 | 19 | 30 | |
| Asparagine | 0.45 | 0.9 | Positive | 133.1 | 87 | 20 | 30 | |
| Glutamine/Lysine | 0.45 | 0.9 | Positive | 147.1 | 84 | 25 | 33 | |
| Methionine | 0.45 | 0.9 | Positive | 150.1 | 133 | 10 | 30 | |
| Histidine | 0.45 | 0.9 | Positive | 156.1 | 110 | 12 | 50 | |
| Phenylalanine | 0.45 | 0.9 | Positive | 166 | 120 | 14 | 34 | |
| Arginine | 0.45 | 0.9 | Positive | 175.1 | 70 | 25 | 47 | |
| Citrulline | 0.45 | 0.9 | Positive | 176.1 | 113 | 17 | 34 | |
| Tyrosine | 0.45 | 0.9 | Positive | 182.1 | 136 | 14 | 37 | |
| Tryptophan | 0.45 | 0.9 | Positive | 205.1 | 188.1 | 16 | 51 | |
| D_4_-Alanine | 0.45 | 0.9 | Positive | 94 | 48 | 10 | 30 | |
| D_8_-Valine | 0.45 | 0.9 | Positive | 126.1 | 80.1 | 10 | 66 | |
| D_2_-Ornithine | 0.45 | 0.9 | Positive | 135.1 | 72 | 19 | 30 | |
| D_3_-Leucine | 0.45 | 0.9 | Positive | 135.1 | 89 | 10 | 30 | |
| D_3_-Methionine | 0.45 | 0.9 | Positive | 153.1 | 136 | 10 | 30 | |
| D_2_-Citrulline | 0.45 | 0.9 | Positive | 178.1 | 115 | 17 | 34 | |
| D_4_-Arginine | 0.45 | 0.9 | Positive | 180.1 | 75 | 25 | 47 | |
| ^13^C-^15^N-Glycine | 0.45 | 0.9 | Positive | 78 | 32 | 10 | 30 | |
| ^13^C5-^15^N-Proline | 0.45 | 0.9 | Positive | 122.1 | 75 | 17 | 32 | |
| ^13^C6-Phenlalanine | 0.45 | 0.9 | Positive | 172 | 126 | 14 | 34 | |
| ^13^C6-Tyrosine | 0.45 | 0.9 | Positive | 188.1 | 142 | 14 | 37 | |

**Supplementary Table 2 The optimized transitions for acylcarnitine.**

| **Compound** | **RT (min)** | **RT Window (min)** | **Polarity** | **Precursor (m/z)** | **Product (m/z)** | **Collision Energy (V)** | **RF Lens (V)** |
| --- | --- | --- | --- | --- | --- | --- | --- |
| C0-Carnitine | 0.45 | 0.9 | Positive | 162.1 | 85 | 23 | 54 |
| C2-Carnitine | 0.45 | 0.9 | Positive | 204.1 | 85 | 21 | 51 |
| C3-Carnitine | 0.45 | 0.9 | Positive | 218.2 | 85 | 21 | 51 |
| C4-Carnitine | 0.45 | 0.9 | Positive | 232.2 | 85 | 22 | 49 |
| C5-Carnitine | 0.45 | 0.9 | Positive | 246.2 | 85 | 23 | 68 |
| C6-Carnitine | 0.45 | 0.9 | Positive | 260.3 | 85 | 23 | 68 |
| C5OH-Carnitine | 0.45 | 0.9 | Positive | 262.2 | 85 | 25 | 49 |
| C5DC-Carnitine | 0.45 | 0.9 | Positive | 276.2 | 85 | 25 | 65 |
| C8:1-Carnitine | 0.45 | 0.9 | Positive | 286.3 | 85 | 24 | 59 |
| C8-Carnitine | 0.45 | 0.9 | Positive | 288.3 | 85 | 24 | 59 |
| C10:2-Carnitine | 0.45 | 0.9 | Positive | 312.3 | 85 | 25 | 65 |
| C10:1-Carnitine | 0.45 | 0.9 | Positive | 314.3 | 85 | 25 | 65 |
| C10-Carnitine | 0.45 | 0.9 | Positive | 316.3 | 85 | 25 | 65 |
| C12:1-Carnitine | 0.45 | 0.9 | Positive | 342.3 | 85 | 27 | 77 |
| C12-Carnitine | 0.45 | 0.9 | Positive | 344.3 | 85 | 27 | 77 |
| C12:1OH-Carnitine | 0.45 | 0.9 | Positive | 358.3 | 85 | 28 | 70 |
| C14:2-Carnitine | 0.45 | 0.9 | Positive | 368.3 | 85 | 28 | 65 |
| C14:1-Carnitine | 0.45 | 0.9 | Positive | 370.3 | 85 | 28 | 65 |
| C14-Carnitine | 0.45 | 0.9 | Positive | 372.3 | 85 | 28 | 65 |
| C14:1OH-Carnitine | 0.45 | 0.9 | Positive | 386.3 | 85 | 30 | 77 |
| C16:1-Carnitine | 0.45 | 0.9 | Positive | 398.3 | 85 | 28 | 74 |
| C16-Carnitine | 0.45 | 0.9 | Positive | 400.3 | 85 | 28 | 74 |
| C18:2-Carnitine | 0.45 | 0.9 | Positive | 424.4 | 85 | 30 | 78 |
| C18:1-Carnitine | 0.45 | 0.9 | Positive | 426.4 | 85 | 30 | 78 |
| C18-Carnitine | 0.45 | 0.9 | Positive | 428.4 | 85 | 30 | 78 |
| D_3_-C18-Carnitine | 0.45 | 0.9 | Positive | 431.4 | 85 | 30 | 78 |
| D_9_-C0-Carnitine | 0.45 | 0.9 | Positive | 171.1 | 85 | 23 | 54 |
| D_3_-C2-Carnitine | 0.45 | 0.9 | Positive | 207.1 | 85 | 21 | 51 |
| D_3_-C3-Carnitine | 0.45 | 0.9 | Positive | 221.2 | 85 | 21 | 51 |
| D_3_-C4-Carnitine | 0.45 | 0.9 | Positive | 235.2 | 85 | 22 | 49 |
| D_9_-C5-Carnitine | 0.45 | 0.9 | Positive | 255.2 | 85 | 23 | 68 |
| D_3_-C5OH-Carnitine | 0.45 | 0.9 | Positive | 265.2 | 85 | 25 | 49 |
| D_3_-C5DC-Carnitine | 0.45 | 0.9 | Positive | 279.2 | 85 | 25 | 65 |
| D_3_-C8-Carnitine | 0.45 | 0.9 | Positive | 291.3 | 85 | 24 | 59 |
| D_9_-C12-Carnitine | 0.45 | 0.9 | Positive | 353.3 | 85 | 27 | 77 |
| D_9_-C14-Carnitine | 0.45 | 0.9 | Positive | 381.3 | 85 | 28 | 65 |
| D_3_-C16-Carnitine | 0.45 | 0.9 | Positive | 403.3 | 85 | 28 | 74 |
| D_3_-C16OH-Carnitine | 0.45 | 0.9 | Positive | 419.3 | 85 | 30 | 77 |

**Supplementary Table 3 The Relationship for Amino Acids Quantification**

| **Compound** | **Internal Standards** |
| --- | --- |
| Glycine | ^13^C-^15^N-Glycine |
| Alanine | D_4_-Alanine |
| Serine | D_8_-Valine |
| Proline | ^13^C5-^15^N-Proline |
| Valine | D_8_-Valine |
| Threonine | D_8_-Valine |
| Pyroglutamic acid | ^13^C5-^15^N-Proline |
| Leucine/Isoleucine | D_3_-Leucine |
| Ornithine | D_2_-Ornithine |
| Asparagine | D_3_-Aspartate |
| Glutamine/Lysine | D_2_-Ornithine |
| Methionine | D_3_-Methionine |
| Histidine | ^13^C5-^15^N-Proline |
| Phenylalanine | ^13^C6-Phenylalanine |
| Arginine | D_4_-Arginine |
| Citrulline | D_2_-Citrulline |
| Tyrosine | ^13^C6-Tyrosine |
| Tryptophan | ^13^C6-Phenylalanine |

**Supplementary Table 4 The Relationship for Acylcarnitines Quantification**

| **Compound** | **Internal Standards** |
| --- | --- |
| C0-Carnitine | D_9_-C0-Carnitine |
| C2-Carnitine | D_3_-C2-Carnitine |
| C3-Carnitine | D_3_-C3-Carnitine |
| C4-Carnitine | D_3_-C4-Carnitine |
| C5-Carnitine | D_9_-C5-Carnitine |
| C6-Carnitine | D_9_-C5-Carnitine |
| C5OH-Carnitine | D_3_-C5OH-Carnitine |
| C5DC-Carnitine | D_3_-C5DC-Carnitine |
| C8:1-Carnitine | D_3_-C8-Carnitine |
| C8-Carnitine | D_3_-C8-Carnitine |
| C10:2-Carnitine | D_3_-C8-Carnitine |
| C10:1-Carnitine | D_3_-C8-Carnitine |
| C10-Carnitine | D_3_-C8-Carnitine |
| C12:1-Carnitine | D_9_-C12-Carnitine |
| C12-Carnitine | D_9_-C12-Carnitine |
| C12:1OH-Carnitine | D_9_-C12-Carnitine |
| C14:2-Carnitine | D_9_-C14-Carnitine |
| C14:1-Carnitine | D_9_-C14-Carnitine |
| C14-Carnitine | D_9_-C14-Carnitine |
| C14:1OH-Carnitine | D_9_-C14-Carnitine |
| C16:1-Carnitine | D_3_-C16-Carnitine |
| C16-Carnitine | D_3_-C16-Carnitine |
| C18:2-Carnitine | D_3_-C18-Carnitine |
| C18:1-Carnitine | D_3_-C18-Carnitine |
| C18-Carnitine | D_3_-C18-Carnitine |
